# A Hepatitis C Virus Infection Model with Time-Varying Drug Effectiveness: Solution and Analysis

**DOI:** 10.1371/journal.pcbi.1003769

**Published:** 2014-08-07

**Authors:** Jessica M. Conway, Alan S. Perelson

**Affiliations:** Theoretical Biology and Biophysics, Los Alamos National Laboratory, Los Alamos, New Mexico, United States of America; Emory University, United States of America

## Abstract

Simple models of therapy for viral diseases such as hepatitis C virus (HCV) or human immunodeficiency virus assume that, once therapy is started, the drug has a constant effectiveness. More realistic models have assumed either that the drug effectiveness depends on the drug concentration or that the effectiveness varies over time. Here a previously introduced varying-effectiveness (VE) model is studied mathematically in the context of HCV infection. We show that while the model is linear, it has no closed-form solution due to the time-varying nature of the effectiveness. We then show that the model can be transformed into a Bessel equation and derive an analytic solution in terms of modified Bessel functions, which are defined as infinite series, with time-varying arguments. Fitting the solution to data from HCV infected patients under therapy has yielded values for the parameters in the model. We show that for biologically realistic parameters, the predicted viral decay on therapy is generally biphasic and resembles that predicted by constant-effectiveness (CE) models. We introduce a general method for determining the time at which the transition between decay phases occurs based on calculating the point of maximum curvature of the viral decay curve. For the parameter regimes of interest, we also find approximate solutions for the VE model and establish the asymptotic behavior of the system. We show that the rate of second phase decay is determined by the death rate of infected cells multiplied by the maximum effectiveness of therapy, whereas the rate of first phase decline depends on multiple parameters including the rate of increase of drug effectiveness with time.

## Author Summary

Fitting simple models of therapy for viral diseases, such as hepatitis C virus (HCV) or human immunodeficiency virus, to patient data has yielded significant insights into the underlying viral dynamics. In general, these models assume that, once therapy is started, the drug has a constant effectiveness. More realistic assumptions are that drug effectiveness either depends directly on the drug concentration or varies over time. Here a previously introduced varying-effectiveness (VE) differential equation model is studied in the context of HCV infection. We show that the previously-unsolved VE model can be transformed into a Bessel equation and derive an analytic solution in terms of modified Bessel functions with time-varying arguments. These analytic solutions can be more readily used to fit the model to patient data than the underlying differential equations. We also find approximate solutions and establish the asymptotic behavior of the system. Typically viral load measurements exhibit a biphasic decline after therapy initiation. We show that the rate of second phase decay is determined by the death rate of infected cells multiplied by the maximum effectiveness of therapy, whereas the rate of first phase decline may depend on multiple parameters, resulting in differing first phase declines across various HCV therapies.

## Introduction

Chronic hepatitis C virus (HCV) infection affects between 150 and 180 million people world-wide and is a major cause of chronic liver disease, cirrhosis and hepatocellular carcinoma. A number of agents have been approved for treating HCV infection including pegylated interferon-alpha (PegIFN) and ribavirin (RBV); the HCV protease inhibitors telaprevir, boceprevir, and simeprevir; and the HCV polymerase inhibitor sofosbuvir [1]. A large number of other agents are being tested in clinical trials [2].

**Figure 1:**
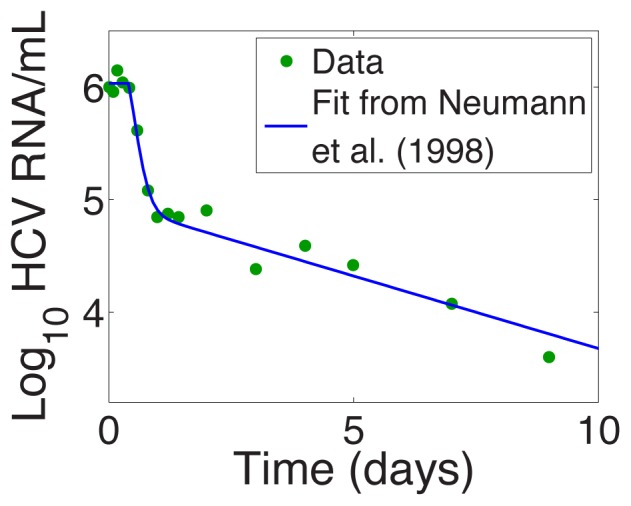
Example of a biphasic decline of HCV, following a short delay, after initiation of interferon-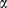 therapy at 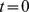. Fit of Neumann et al. model (solid line) to data for Patient 1E (dots) from [3].

An early model of HCV infection and treatment developed by Neumann et al. [3] showed that the effectiveness of antiviral therapy in blocking HCV production from infected cells could be estimated from the kinetics and extent of viral decline during the first few days of therapy. Neumann et al. [3] also showed that if plasma HCV RNA levels were measured frequently after treatment initiation with interferon one observed a biphasic decline after a short delay when the logarithm of HCV RNA/ml was plotted versus time on treatment (Fig. 1). This type of biphasic decline has now been observed with many different types of HCV treatments including those employing PegIFN and RBV, and a variety of HCV protease and polymerase inhibitors [4]–[10].

The Neumann et al. model [3] assumed that there was delay before interferon became active followed by a period in which it had constant effectiveness. Under reasonable assumptions, this leads to a model described by a set of linear, constant coefficient, ordinary differential equation that can easily be solved [3]. Models, such as that of Neumann et al., in which the drug effectiveness is constant or constant after a delay have been called constant effectiveness (CE) models [11], [12]. In the case of interferon therapy we now know that the delay is caused by pharmacokinetics of the drug as well as the time needed for the drug to bind cell surface interferon receptors and cause upregulation of interferon stimulated genes, whose gene products then lead to reduced viral replication.

For pegylated interferon, which is approved for once weekly dosing, the pharmacokinetics of the drug lead to a loss of effectiveness towards the end of the dosing interval in many patients [13], [14]. To account for this, a combined pharmacokinetic/viral kinetic model was introduced by Powers et al. [13] and fit to both drug concentration and HCV RNA data by Talal et al. [14]. However, in most clinical studies drug concentration data is not available for each patient. A phenomenological time-varying effectiveness (VE) model was therefore introduced by Shudo et al. [11], [12] and studied numerically. Guedj at al. [6] studying the effects of the HCV protease inhibitor telaprevir on viral decay kinetics showed that a VE model fit clinical data better than a CE model as assessed by the Akaike Information criterion, which allows one to compare the ability of models with different numbers of parameters to fit data [15].

This study was followed by two others by Guedj et al. using VE models to analyze the HCV RNA decay kinetics observed with the nucleoside polymerase inhibitor mericitabine [16], and with the HCV nucleotide polymerase inhibitors sofosbuvir and GS-0938 [17]. In these cases, the VE model accounted for the fact that these drugs need to be triphosphorylated intracellularly to become active [18]. More recently, Canini et al. [19] used a VE model to analyze the viral kinetics seen in a different set of patients treated with the drug silibinin, which appears to have activity as both a polymerase and entry inhibitor [20], [21]. In all of these studies employing VE models, numerical methods were used to solve the time-varying equations. Here, we show how a previously used and prototypic VE model can be analyzed mathematically. We obtain an analytic solution to the time-varying problem in terms of modified Bessel functions, and a set of approximate solutions involving exponential decay functions.

## Models

We model HCV viral dynamics at the initiation of treatment by modifying the standard constant effectiveness viral dynamic model of Neumann et al. [3]. For infected cells, 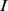, and viral load, 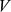, the model differential equations are 
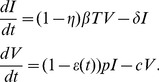
(1)


We assume the number of target cells, 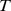, is constant and takes on its pre-therapy steady-state value, 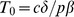. This is an approximation that is commonly made when analyzing clinical trial data obtained over a period of one or two weeks. In the case of Neumann et al. [3], it was used to analyze data collected over two weeks.

In the model given by equation (1), target cells are infected by virus, 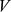, with mass-action infectivity 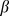. Infected cells die at rate 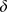 per cell and virus clears at rate 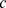 per virion. The infection process may be hampered by drug treatment; the efficacy of treatment in blocking infection is given by 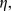 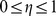. Infected cells produce virus at rate 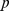 per cell. Drug treatment may also interfere with viral production, with efficacy 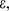 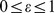. In the constant effectiveness (CE) model the drug efficacy is assumed to be constant, 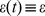. In this case the solution for the viral load dynamics from (1) is 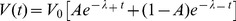
(2)where 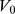 is the viral load at 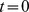, 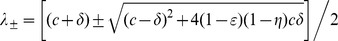, and 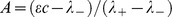 [3], [21]. Here we assume the drug efficacy in blocking viral production, 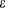, is time dependent, i.e. 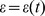, with a build-up of activity to a maximum 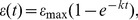
(3)where 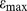 is the maximum drug efficacy obtained with the concentration of drug used and the exponential scale 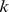 determines the speed at which the drug efficacy reaches its maximum (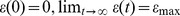). In principle, the effectiveness of treatment in blocking infection, 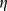, could also be time dependent. Here we have chosen to ignore this possibility as no published data is available to guide such modeling efforts.

At treatment initiation (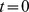) we assume the system is in steady state. Let the initial viral load, i.e., pre-treatment viral load set-point, be given by 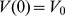. Since we assume that pre-treatment 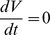, then 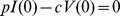 and 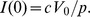 Further, 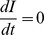, so that 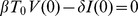. Since 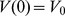 and 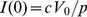, 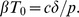


Substituting for 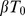, our system becomes 
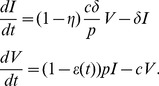
(4)


Now let 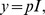 and for notational convenience let 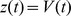 and 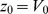


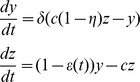
with initial conditions 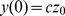 and 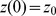. In the next section we will find an analytic solution for our model using this formulation.

## Results

### Analytic solution

We are interested in solving the system of ODEs 
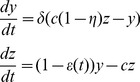
(5)with initial conditions 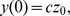 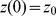, where 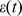 is the time-dependent drug efficacy 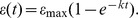 Assume that 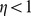; we treat the 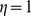 case separately below. We can re-write this as a linear system, 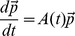
where 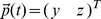 and 
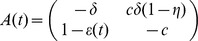
. 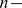
dimensional systems for 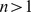 of the form 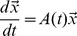 have solutions, Magnus expansions, that are infinite series, which only collapse to a single term giving a closed for solution if, for any 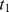, 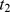, 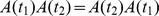 [22], [23]. Since 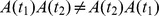, our system of equations (5) has no closed form solution.

However we can still recover a solution. We begin by writing the system (5) as a second-order differential equation. First, let 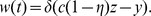 Then 
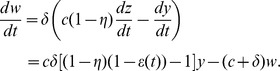



Our system of equations (5) then becomes 
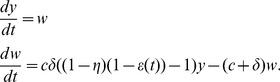
(6)


Since 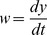, 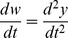 and from (6) we recover the second order equation corresponding to the system of ODEs (5), 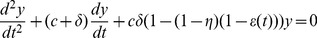
(7)with initial conditions 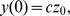 
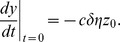


We now employ some convenient changes in the dependent and independent variables. Let 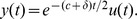 Then (7) becomes 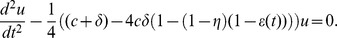



Then let 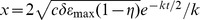 (recall that 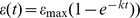, so that 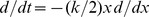 and 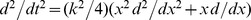, to obtain 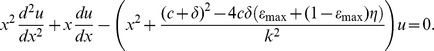



Finally let 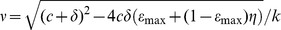, to simplify the equation 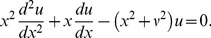
(8)


Equation (8) is the modified Bessel differential equation [24], with solutions 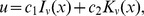
where 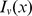 and 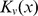 are the modified Bessel functions of the first- and second-kind of order 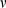. As they represent infinite series, Bessel functions are not closed-form solutions. Note that the order 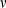 is real: since 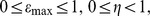 the factor 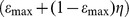 varies between 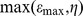 and 1. Thus 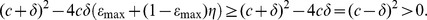



Then since 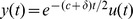, the solution of equation (7) is 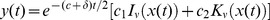
(9)where 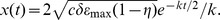


We can use the solution (9) and the initial conditions 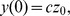 
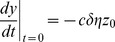
, to solve for the constants 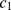, 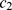. Let 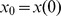 and note that 
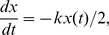
 so that 
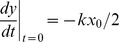
. Then, noting that 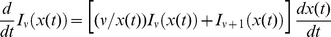 and 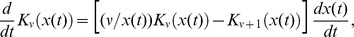 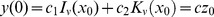


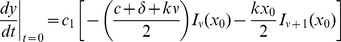


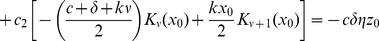
and 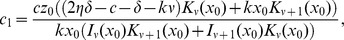


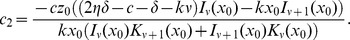



Since 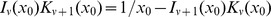 [24] the constants can be written more simply as 
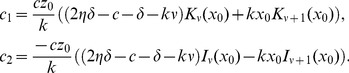
(10)


To recover 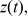 recall that 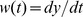 and 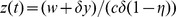. Therefore 
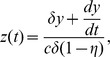
with 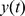 given by (9), 
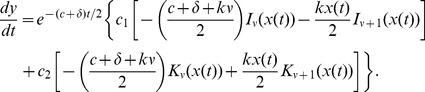



Thus the viral load, 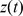, is given by 
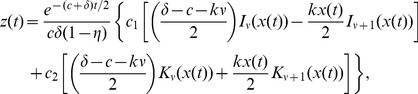
(11)where 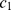, 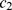 are given by (10), 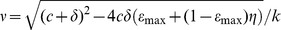, and 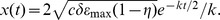


### Solution for general varying effectiveness model

The varying effectiveness model employed above is a simplification of the more general time-varying effectiveness model, 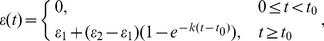
(12)which has been useful in cases where the viral load shows no measurable decay until time 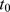 [6], [16]. Since at low values of the effectiveness no change in viral load may be discerned due to low assay sensitivity and noise, one assumes the effectiveness has value 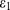 at the time viral load declines become measurable. With 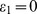, 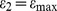, and 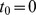 we recover the simpler form, equation (3). The analytic solution for this more general VE model can be found following the approach described above, yielding 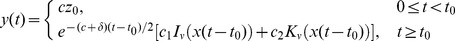


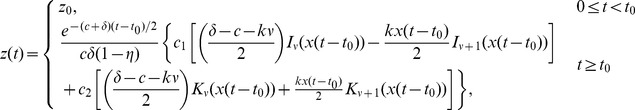
where 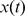 is now given by 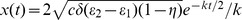, and the order 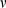 is 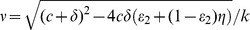 (as before with 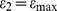). The constants 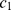, 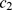 are still given by (10) but with 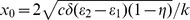 instead.

### Analytic solution for 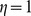


The parameter 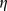, 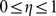, represents the drug's effectiveness in interfering with new cell infection with 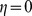 indicating no efficacy and 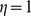 indicating perfect efficacy. The analytic solution (11) assumes 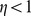. Perfect drug efficacy, 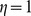, is not a biologically reasonable assumption. However, for drugs or drug combinations with very high effectiveness in blocking viral production, viral loads fall profoundly after therapy initiation and new cell infections become rare. Under such circumstances, the solution with 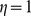 (i.e. no new infections after therapy is initiated) may be a reasonable approximate model [25], [26].

Given 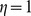 the equation for infected cells, 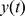, from (5) becomes 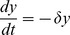. With initial condition 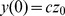 the solution is 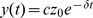. Then the equation for viral load, 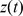, from (5) becomes 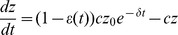
with initial condition 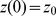. We can re-write this equation, using an integrating factor, as 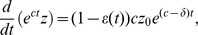
where 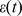 is given by (3). Integrating, we obtain 
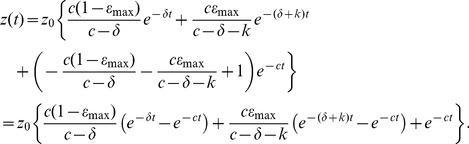
(13)


For the more general varying effectiveness model given by (12), the analytic solution given 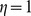 for 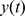 and the viral load, 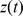, is 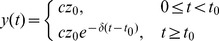


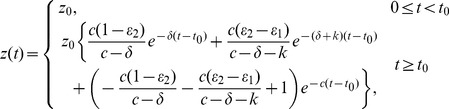



We note for both VE models there exist three time-scales given by the exponential decay rates 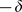, 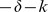, and 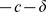.

### Transition time calculation

**Figure 2:**
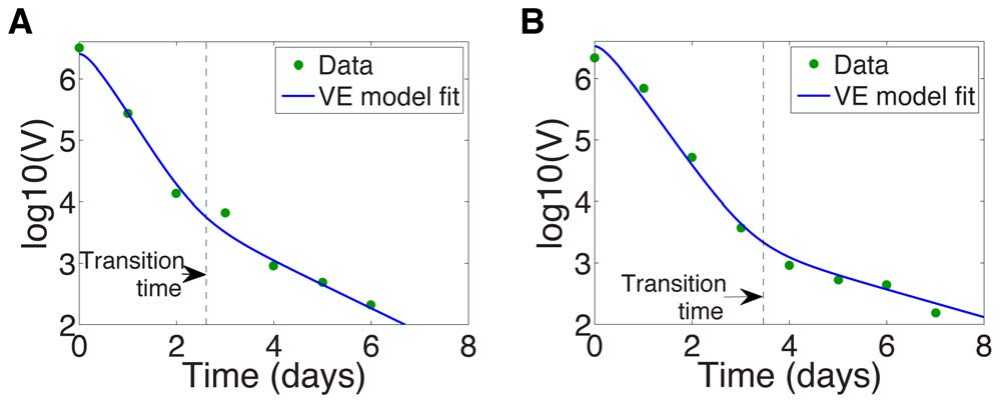
HCV viral load undergoes biphasic decay upon initiation of silibinin treatment at time 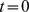. The transition time between the first and second phases, 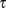, is calculated by maximizing the curvature 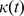 in equation (14), and is marked by a vertical dashed line. VE model fit of Canini et al. [19] (solid line) and HCV viral load data (dots) for (a) Patient 46, with transition time 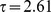 days, and for (b) Patient 48, with transition time 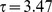 days.

As noted before, in biologically reasonable parameter regimes this model predicts that, after initiation of antiviral therapy, viral load usually undergoes a biphasic decay, consistent with observations on many different types of HCV treatments [3], [6], [16]. Examples are given in Figs. 1 and 2, which show the log of the viral load after treatment initiation at time 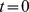. The transition time between the fast- and slow- decay phases, marked by a dashed line in Fig. 2 is also of clinical interest. For example, with silibinin treatment the transition time has been shown to vary with the patient's disease progression state (chronic HCV, compensated/decompensated cirrhosis) [19].

At the transition time, the viral load curve has maximal curvature (c.f. Fig. 2). The curvature of the plane curve 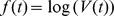, 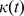, is given by 
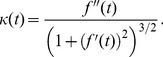
(14)


[27]. Therefore, to calculate the transition time, 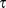, we calculate the time when the curvature 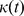 is maximized. To do this we numerically solve 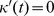 using the analytic solution for 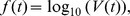 where 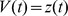 from (11).

We can use this curvature-based approach to analytically calculate the transition time for the CE model (2). Maximizing the curvature 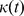 (14) for the CE model (2), the transition time 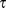 is the solution of 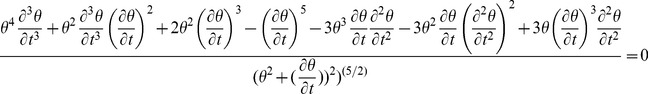
(15)for 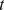 with 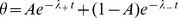 (in (2), 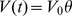). The solution of (15) is lengthy and is not included here for brevity. Supporting Fig. S1 shows patient data and model fits from [3] with the transition times marked.

### Parameters: Typical behaviors of different drug classes

The model (1), with varying drug efficacy (12), has been used to investigate a number of drug treatments for HCV. Here we discuss therapy with four drugs: the protease inhibitors (PIs) telaprevir and danoprevir, the nucleoside polymerase inhibitor (NPI) mericitabine, and silibinin, a compound extracted from milk thistle seed. Silibinin is intriguing because, in addition to interfering with viral production as with the PIs and NPIs, it also appears to have some cell infection interference capabilities [21], [28]. This additional capability is modeled by the 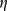 term in (12), 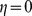 for telaprevir, danoprevir, mericitabine, and sofosbuvir. Table 1 gives published estimates for model and drug parameters, obtained by fitting VE models to patient data, under the different treatment types, and when available different dosing regimens. The therapy durations were all two weeks or less so the assumption of a constant level of target cells was made in the primary publications from which the parameter estimates were obtained.

**Table 1:** Model parameter estimates obtained for different drug treatments of chronic HCV.

Treatment	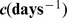	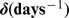	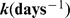	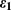	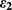	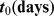	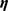	Trial Dur.	Source
Telaprevir	13.4	0.58	2.86	0.974	0.999	0.10	n/a	2.5 days	[6]
Mericitabine, qd 750 mg	6*	0.023	1.06 (flat)	0.38	0.86	0.37	n/a	2 weeks	[16]
			0.23 (non-flat)						
Mericitabine, qd 1500 mg	6*	0.023	1.06 (flat)	0.38	0.94	0.37	n/a	2 weeks	[16]
			0.23 (non-flat)						
Mericitabine, bid 750 mg	6*	0.023	2.03 (flat)	0.38	0.98	0.37	n/a	2 weeks	[16]
			0.43 (non-flat)						
Mericitabine, bid 1500 mg	6*	0.023	2.03 (flat)	0.38	0.998	0.37	n/a	2 weeks	[16]
			0.43 (non-flat)						
Silibinin	6*	0.62	2.12	n/a	0.861	n/a	6**	7 days	[19]
Danoprevir, bid 100 mg	7.25	0.184	29.1	n/a	0.973	n/a	n/a	13 days	***
Danoprevir, bid 200 mg	7.25	0.184	29.1	n/a	0.985	n/a	n/a	13 days	***
Danoprevir, bid 300 mg	7.25	0.184	29.1	n/a	0.99	n/a	n/a	13 days	***
Sofosbuvir	5.76	0.53	8.12	n/a	0.998	n/a	n/a	7 days	***

Model parameter 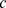 gives the viral clearance rate, 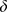 the infected hepatocyte death rate, 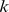 gives the exponential scale at which the drug reaches its maximum value 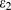 from its minimum value 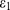, 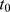 gives the delay in the drug activity, and 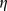 gives the efficacy of treatment in blocking new cell infection.

*Clearance rate 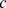 fixed at 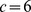 days^−1^ from [3], not estimated.

**Efficacy of treatment in blocking new cell infection 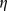 fixed at 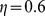 from [16], not estimated.

***Unpublished.

**Notes**: (**i**) In fitting viral load data, authors investigating telaprevir and mericitabine used the more general VE model (12), while those investigating silibinin, danoprevir, and sofosbuvir used the simple VE model (3) with 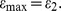 Efficacy of treatment in blocking new cell infection 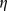 was only used in the silibinin model (effectively 0 in other models). (**ii**) qd  =  daily dosing, bid =  bi-daily dosing. (**iii**) Parameter estimates derive from HCV treatment studies on patients who were treatment naïve, except in the case of mericitabine, where all patients were interferon non-responders.

In the following section we analyze the analytic solution of (1), given by (11), in order to gain some insight into long- and short-term behavior. Knowledge of the magnitude and relative size of model parameters is very helpful in such analyses. Table 1 reveals that estimates from different studies are not always consistent: observe that estimates for the hepatocyte death rate 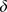 are an order of magnitude smaller for the mericitabine fits relative to the telaprevir, danoprevir, silibinin, and sofosbuvir. This discrepancy arises from the patient data used in model fitting: patients on telaprevir, danoprevir, silibinin, and sofosbuvir were treatment naïve, while patients put on mericitabine had already experienced PegIFN and RBV treatment failure. Regardless, we note that

1. Final drug efficacy is quite high, 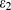 close to 1 (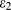 in the general VE model (12) is equivalent to 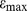 in the simpler VE model (12)).
2. Viral clearance rate 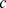 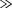 infected cell death rate 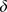 (also the case for the constant effectiveness model [3]
3. The rate of effectiveness increase 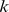 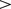 infected cell death rate 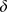

across all cases. We will exploit these relationships in the asymptotic analysis below.

We also note that the rate of effectiveness increase, 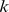, can vary by orders of magnitude between drug types. For example, 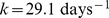 in the case of danoprevir treatment, 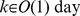 for telaprevir and silibinin treatments. Analysis of viral dynamics in patients on mericitabine revealed two distinct biphasic viral curve types across patients: the first with a flat second phase, the second with a non-flat (decaying) second phase [16]. The covariate distinguishing these two groups remains unclear. But the fits suggest the distinction lies with the parameter 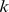, since non-flat second phases have 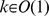 and flat second phase patients have 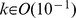 [16]. In the following analysis we will consider 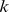 across orders of magnitude.

### Approximations to the analytic solution; short- and long-time behavior

The argument of the modified Bessel functions in (11) is 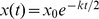, where 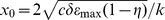. For small 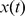 we can use the following approximations [24] for modified Bessel functions with small argument 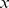, 
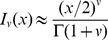
and 
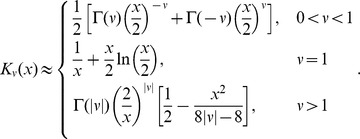



We will neglect the 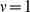 case since it is highly unlikely that a set of realistic parameters will yield 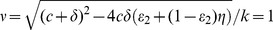 exactly. The approximation for 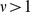 is actually valid for 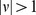 but since 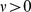 we can drop the absolute value signs 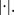 Since 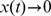 monotonically as 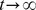 we expect the approximations to hold for long times. Note that 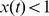 for 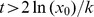 and 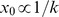, so we anticipate that the approximations are appropriate even at short times for sufficiently large 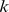. Applying the approximations to (11), 
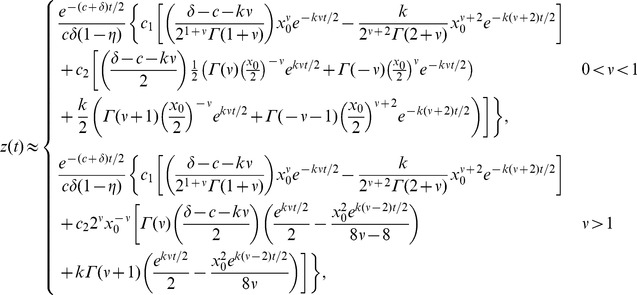
(16)


**Figure 3:**
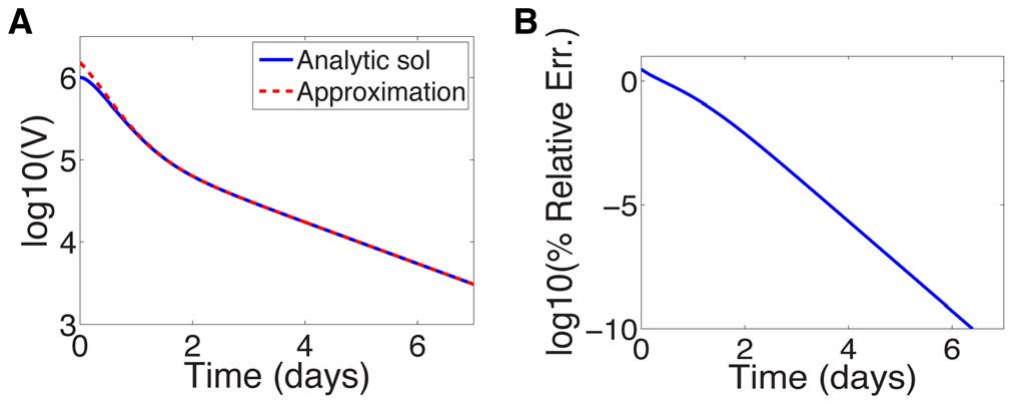
Approximate and analytic solution of VE model. (a) Comparison of analytic solution (equation (11)) and the approximation (equation (16)) assuming sibilinin treatment (see Table 1 for parameters) and initial viral load of 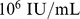. (b) Relative error in 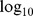 of approximation.

The order 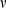 for each treatment regimen shown in Table 1 is given in Supporting Table S1 for reference. Fig. 3a shows a comparison between the approximation (16) and the analytic solution (11) for parameters characterizing silibinin (Table 1). Near 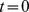 the error in the log of the approximation is 5% and improves significantly with increasing 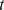 (see Fig. 3b). This improvement is not surprising: the approximations are for small 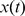 and 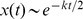 grows smaller with increasing 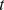. Therefore we can use the approximation to gain insight into the long-time behavior.

We may also be able to use the approximation to gain some insight into the short-time behavior; although the errors near 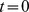 are not negligible, the approximation remains within the right order of magnitude, and away from 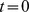 the slope of the solutions appear similar with these parameters, see Fig. 3a. The approximation does not however capture the shoulder in the analytic solution near 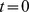.

#### Long-time behavior

From (16) we note three (for 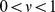) or four (for 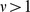) distinct exponential decay rates: 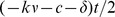, 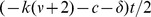, 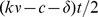, and, for 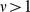, 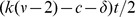. Since all parameters are positive and 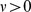, the slowest decay corresponds to 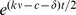. Recall that 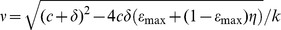. If the maximum drug efficacy, 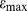, is close to 1, 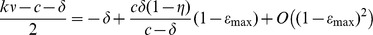



In the long term, viral load decays approximately as 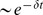. Further, if 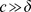 as it is for HCV (cf. Table 1) then 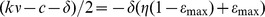, which is 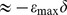 for 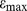 close to 1. Not surprisingly, this is equivalent to the long-term decay rate 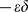 previously predicted by the CE model using similar parameter values [3].

#### Short-time behavior

Away from 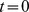 the approximate solution and analytic solution show good matching, and have similar first-phase slopes (Fig. 3). The approximation may therefore give us some insight into the first-phase decay rate. Let 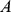 represent the term in equation (16) that contains 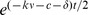, 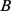 the term containing 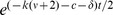, 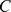 the term containing 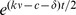, and 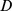 the term containing 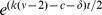. 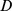 is only present in the approximation for 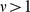. As before, since 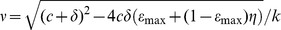 and 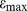 is near 1, to leading order the exponential decay rates are 
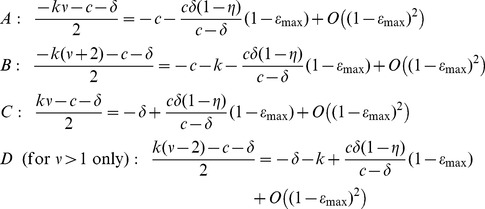
(17)


**Figure 4:**
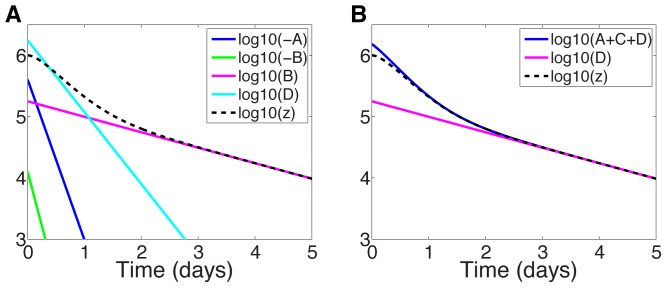
Different exponential terms in approximate solution (16) compared with the exact solution and for silibinin treatment parameters, for which 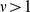 (see Table 1). (a) Exponential terms from (16) plotted separately. (b) Exponential terms from (16) plotted in combined form.

Fig. 4 shows the terms 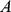, 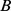, 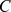, and 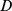 plotted against time for sibilinin parameters (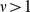; see Table 1), compared to the exact solution (11). Note that for long times, 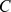 dominates (exponential decay rate 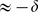), as discussed above. For short times (before the transition between phases at 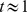) the dominant decay rate is not so obvious. It is somewhat represented by 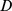 (exponential decay rate 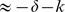), as shown in Fig. 4a. But only when we add the 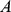 (exponential decay rate 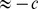) and 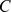 (exponential decay rate 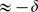) terms do we obtain a reasonable approximation (Fig. 4b). The first phase decay time scale is therefore set by 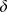, 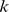 and 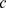, with the initial shoulder not captured by the approximation.

**Figure 5:**
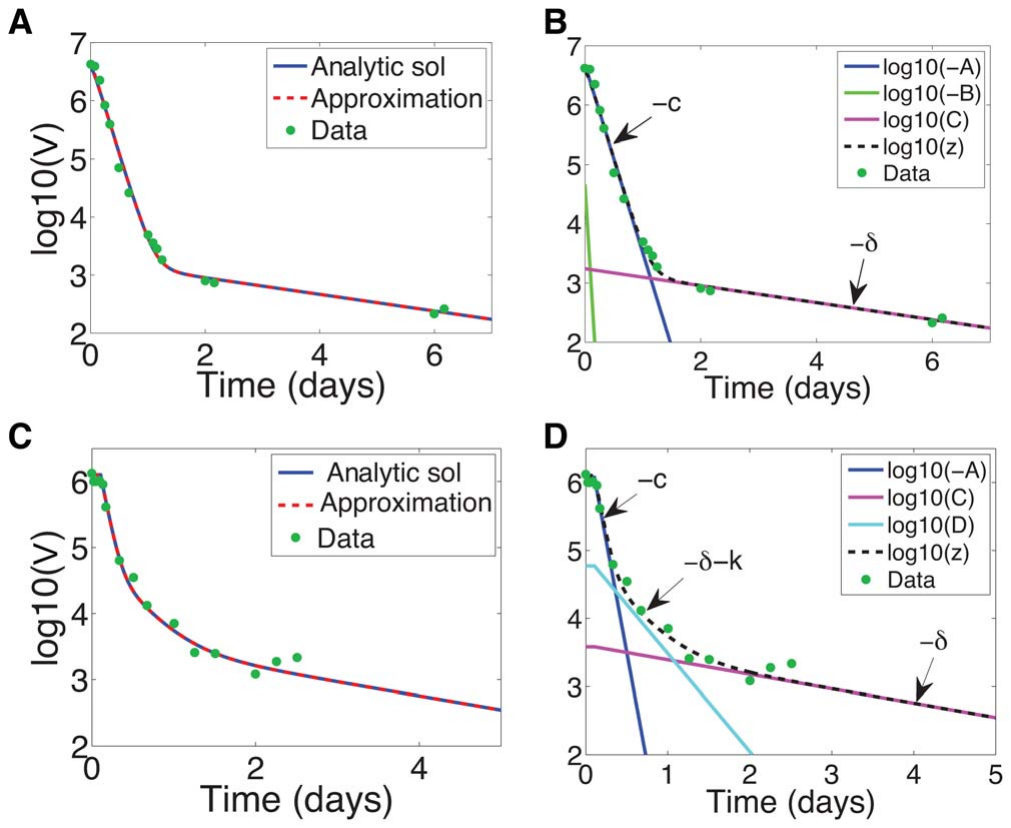
Approximate and analytic solution of the VE model under danoprevir (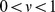) or telaprevir (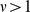) treatment with patient data. (a,c) Approximate solution (16) compared to the analytic solution (11) for (a) danoprevir or (c) telaprevir treatment. (b,d) Different exponential terms in approximate solution compared with the exact solution, with decay phases indicated, for (b) danoprevir or (d) telaprevir treatment. Danoprevir treatment: data from patient 04-94XD (dosing 200 mg tid) in [25] with associated parameter estimates for VE model 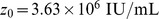, 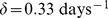, 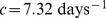, 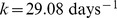, 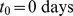, 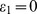, and 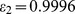 [unpublished]. Telaprevir treatment: data from patient 6 in [6] with associated parameter estimates 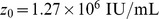, 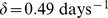, 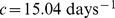, 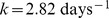, 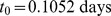, 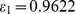, and 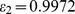 [6].

For 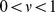, Figs. 5a,b show 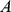, 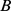, and 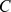 plotted versus time for danoprevir parameters (see Table 1), compared to the exact solution (11). Note that for long times, again, 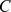 dominates (exponential decay rate 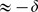). For short times (before the transition between phases at 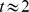) the dominant decay rate is given by 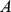 (exponential decay rate 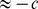). In this case the first phase decay time scale is therefore set by 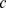. This is not entirely surprising: as 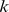 grows large the VE model increasingly resembles the CE model, and for the CE model the first phase time scale is given by 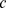 and the second by 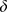 [3], Note that, while it's not obvious from the log-scale in Figs. 5a,b, the initial shoulder, which is now very short, is still not captured by the approximation.

**Figure 6:**
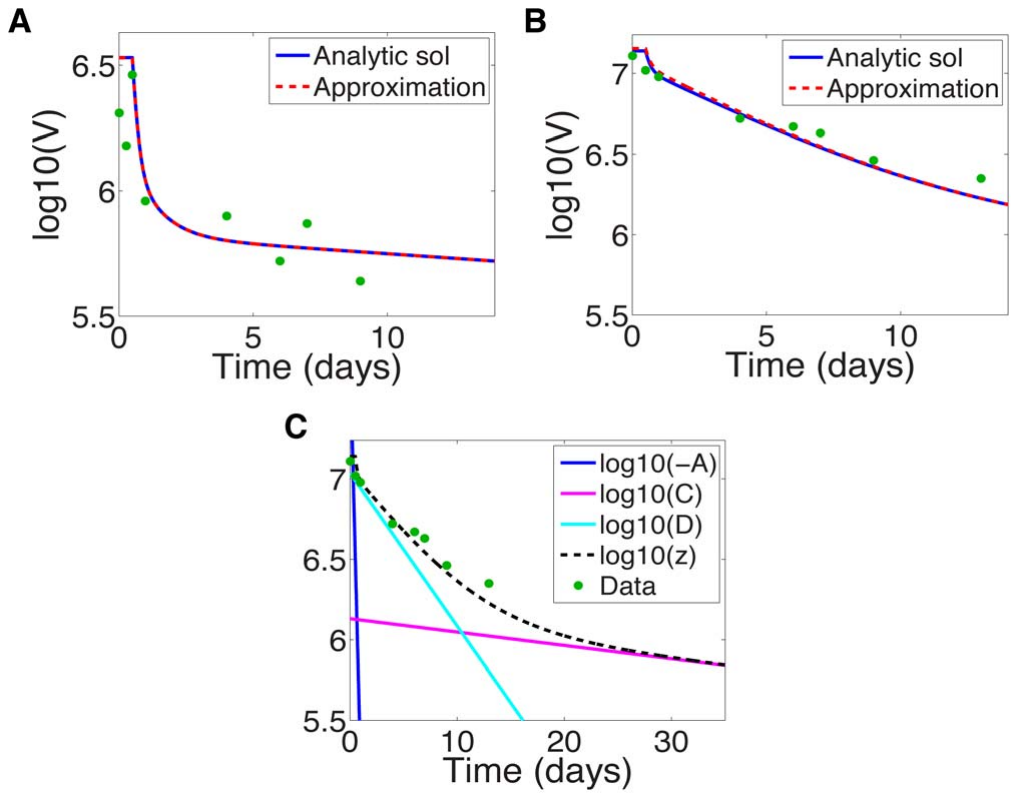
Approximation to viral dynamics compared to exact dynamics under mericitabine treatment, 750 mg qd, 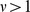. (a) For patient 92102 from [16], characterized as “flat”. (b) For patient 92103 from [16], characterized as “non-flat”. (c) Different exponential terms in approximate solution (16) compared with the exact solution for patient 92103, characterized as “non-flat”. Parameter estimates from [16]: For patient 92102, 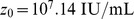, 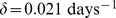, 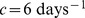, 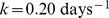, 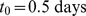, 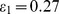, and 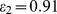; for patient 92103, 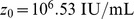, 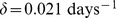, 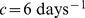, 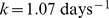, 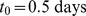, 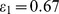, and 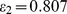.

Interestingly, examining the dynamics under telaprevir treatment reveals that there are arguably three phases, see Figs. 5c,d. Note from Fig. 5c that the full approximation (16) is very good. As shown in Fig. 5d, the initial dynamics are well captured by 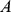 (exponential decay rate 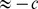), and the long-term dynamics - as always - by 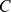 (exponential decay rate 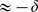). But between the two there is a decay well described by 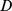 (exponential decay rate 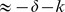). Numerically for telaprevir treatment these three exponential decay rates are 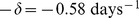, 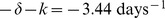, and 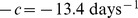 (see Table 1), separated by an order of magnitude, so it is not surprising that we discern three phases. We similarly discern three predicted phases under mericitabine treatment in patients characterized as “non-flat”, see Figs. 6b,c.

When there are more than two decay phases, for example as shown in Figs. 5c,d, the transition time calculation becomes more complicated. We compute the transition time as the time when the curvature 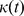 of the log-viral load decay curve is maximized, treating the curvature maximization problem as a non-linear root finding problem, i.e. solving 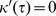 for 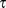. Multiple phase decay would yield multiple transition time solutions, with transition times indicating transition between decay regimes (e.g. under telaprevir treatment, dominance of 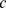, 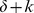, or 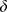, as in Figs. 5c,d). Unfortunately, if the intermediate phase is not sufficiently distinct from the decay phases preceding and following it, the viral load decay may become too rounded, and our method may not give correct transition times.

The approximations (16) are valid for 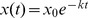 small, and therefore we expect the approximations to improve for smaller 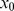 and larger 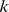 (so that 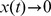 faster). For example, the approximation under telaprevir treatment is better than that for silibinin treatment (compare Fig. 3a and 5c); for telaprevir, 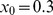 and 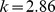, while for silibinin, 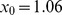 and 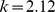. In the next section we will look at a series expansion of the exact solution to show what may be missing from these approximations.

### Series expansions of exact solution

The modified Bessel functions are infinite series and can be expressed as follows: 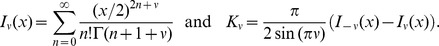



For simplicity let 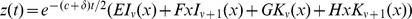 with 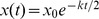 (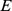, 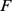, 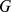, and 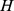 are the constant coefficients in equation (11)). Using the series expressions for Bessel functions we can re-write (11) as a series of exponential functions, 
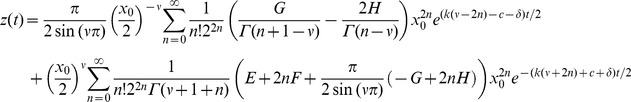
(18)


Since 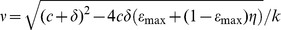 and the maximum drug efficacy, 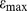, is close to 1, the exponents can be written as 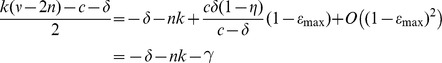


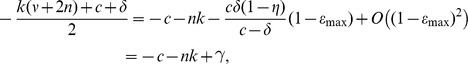




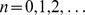
, where 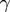 is the sum of the remaining terms in the Taylor series expansion, 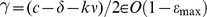. We can re-write the series expansion for the exact solution (11) as 
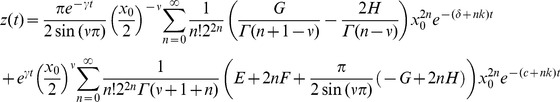
(19)

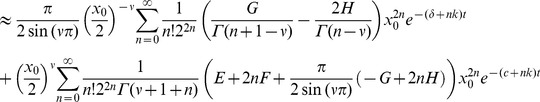
(20)


since 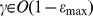. As 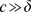, 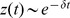 as 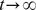. Short term behavior is more difficult to discern as it depends on the magnitude of 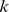. We can use this series expansion to evaluate parameter regimes within which the approximation (16) is valid with regards to the parameter 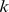.

The exact solution (19) depends on the exponential decay rates 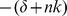 and 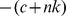 where 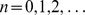. The approximation (16) for small argument 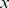 depends on the exponential decay rates 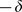, 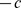, 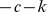, and 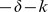 (the latter in the 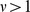 case only). For these to be the most slowly decaying rates of the exact solution (19), 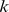 is constrained (recall 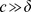):

- For 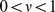, 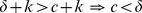 which is never satisfied.
- For 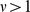, 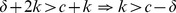, which is not satisfied for any treatment regimen (see Table 1).

However, from Figs. 3, 5a, 5c, and 6a,b, it is clear that in spite of the fact that 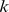 does not satisfy the relevant condition, the approximations can be reasonably good. Direct examination of the numerical values of parameters reveals the source: the relative value of 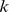. A summary of how the approximations behave with 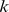 is given in Table 2.

**Table 2:** Goodness of approximation, Eq. (16), for ranges in the parameter 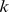.

Case	Treatment types	Interpretation of phases
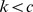	Telaprevir	The approximation gives a reasonable fit but misses the shoulder. Short-time behavior is given by a combination of 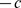 and 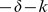 decay rates, with long-term behavior given by the 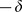 decay rate. For 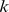 sufficiently small, viral load decay appears tri-phasic, with 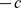 , 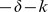 as two separate phases, for example for telaprevir and mericitabine in “flat” patients (see Figures 5c and 6b). Otherwise viral load decay appears biphasic, as with silibinin, see Figure 3a. Shoulder fit improved with inclusion of additional terms, see Figure 7.
	Mericitabine, qd 750 mg, flat	
	Mericitabine, qd 750 mg, non-flat	
	Mericitabine, qd 1500 mg, flat	
	Mericitabine, qd 1500 mg, non-flat	
	Mericitabine, bid 750 mg, flat	
	Mericitabine, bid 750 mg, non-flat	
	Mericitabine, bid 1500 mg, flat	
	Mericitabine, bid 1500 mg, non-flat	
	Silibinin	
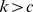	Danoprevir, 100 mg	The approximation gives a good fit. First phase decay rate 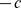 , second phase decay rate 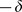 . In general if 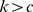 time scales separate sufficiently so that fits will be good, for the example of danoprevir see Figure 5a.
	Danoprevir, 200 mg	
	Danoprevir, 300 mg	
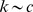	Sofosbuvir	The approximation gives a poor fit for short-time behavior, see Supporting Fig. S2. In this case the time scales separate poorly. To capture any short term behavior, more terms from the series solution (19) are required.

**Figure 7:**
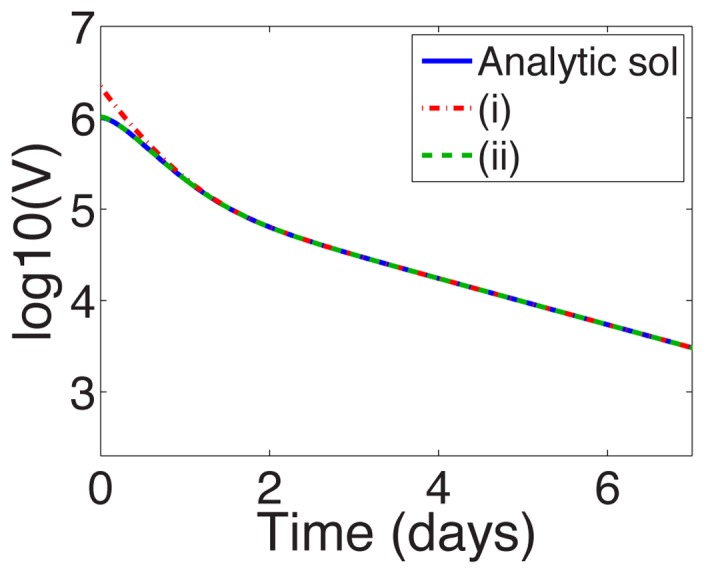
Truncated series solutions for the VE model compared with the exact solution (11) under silibinin treatment (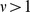; see Table 1 for parameters). Legend: (i) Series terms with exponents 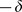, 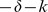, 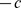, and 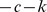 terms, included in the approximation (16), from the series solution (19); (ii) Series terms with exponents from (i) and also the 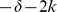 and 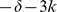 terms missing from the approximation.

## Discussion

Viral dynamic models of infection and treatment have frequently described the effect of therapy by a parameter, 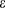, the effectiveness of therapy, where 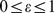. For example, if therapy blocks production of new virus from infected cells, then the rate of production 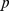 under therapy is modeled as 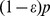, so that when the drug is 100% effective, 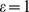 and no viral production occurs. This type of formulation has been used in modeling treatment for HIV [29], [30], HBV [31]–[33], HCV [3], [7], and influenza [34]. However, the effectiveness of a drug frequently depends on its concentration and more complex models incorporating drug pharmacokinetics (PK) and drug pharmacodynamics (PD) have also made their way into viral dynamic modeling [13], [14], [17], [35]–[37].

In many cases, drug concentrations are not measured and detailed PK/PD modeling cannot be performed. Nonetheless, it is clear that variations in time occur in drug concentration. Further, drug activity can also be time-dependent particular when the drug given is a “pro-drug” that needs to be metabolized into an active compound. For example, nucleoside or nucleotide reverse transcriptase inhibitors and polymerase inhibitors need to phosphorylated intracellularly to become active inhibitors [38], [39]. One mechanism to account for time dependent changes in drug activity is to assume that the drug effectiveness, 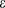, rather than being constant is time dependent. Here we have studied in detail an HCV model in which the effectiveness increases with time to a maximum, assuming either 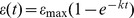 or a more general form 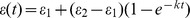, where 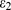 plays the role of 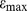. We showed that the HCV model with time-varying effectiveness, previously used in [6], [11], [12], [16], [17], can be solved explicitly in terms of modified Bessel functions.

One reason the model equations can be solved analytically is that the assumption 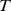 = constant is made, linearizing the mass-action infection term 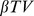. The assumption of constant 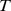 has typically been made when short-term (2 week or less) clinical trials are examined. However, the obtained solution may be more general, particularly for direct-acting antivirals. When therapy is very potent so the viral load rapidly decays many logs during the first days of therapy, as seen for example with daclatasvir, where 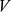 decays 3 logs in the first 12 hrs of therapy [26], the term 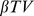 no longer significantly influences the dynamics. Thus, after a very brief transient, whether 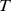 is constant or not may have no practical effect on the underlying viral dynamics. Guedj et al [26] showed this to be the case for daclatasvir by finding an extremely accurate approximate solution to the viral dynamic model they used by assuming there were no new infections after therapy started, i.e. that 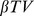 = 0.

Plotting the solution for the viral load, 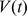, on a logscale we noticed that the virus appeared to decay with time on treatment in a biphasic manner for certain parameters of interest. Such biphasic declines have been observed in HCV patients treated with many different therapies and the lengths of each phase and the rates of decay during each phase are of biological interest [19]. We characterized the transition between phases as the point of maximum curvature in the solution, which can be computed from the solution. However, in order to ascertain the dominant decay rates during these two observable phases, we wanted to find approximations in terms of exponentials. While the model differential equations are sufficient to fit the data, the analysis that permits us to characterize the decay phases is only possible given the analytic solution. To this end, we examined classic approximations to Bessel functions as well as series expansions and showed that the long-time decay is dominated by the rate of loss of HCV-infected cells, 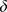, as had previously been shown in constant effectiveness models [3]. This is not surprising since at long times, 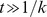, the drug effectiveness approaches a constant value, its maximum. At short times, the constant effectiveness model predicts the rate of viral decay is governed by the rate of viral clearance, 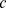. Here with the variable-effectiveness model we find that this need not be the case and more complex relationships between 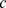, 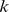 and 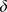 govern the short-term behavior. Using parameters estimated in previously published drug-treatment studies we showed how different combinations of parameters govern the short-term decay for different drug therapies. For example, when 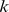 is large compared to 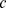 and 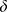, as had been previously found for the HCV protease inhibitor danoprevir, the effectiveness rapidly approaches a constant and the first phase decline is essentially governed by 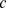 as in the constant effectiveness model. However, when 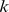 is comparable to or small than 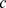 this is no longer the case and 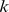 then plays a role in determining the first phase decay. We discovered for parameters governing the HCV protease inhibitor telaprevir, where 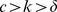 that three distinct exponential phases appeared to govern the viral load decay, with rates of 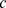, 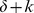, and 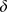. Viral decline under telaprevir treatment had been previously described as biphasic [6]; it is only through the approximations to the analytic solution that the middle, 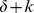, phase was revealed.

The model upon which we based our analysis, while derived for HCV, applies to a number of viral infections. For example, essentially the same model can be used for protease inhibitor treatment of HIV, since HIV protease inhibitors reduce the rate of production of infectious virus. Similarly, neuraminidase inhibitors used to treat influenza A virus infection also reduce the rate of production of infectious virus and again our results would apply. HIV reverse transcriptase inhibitors act to block infection. To analyze this situation would require a generalizationq of our current model in which the parameter 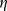 rather than being constant was allowed to be time-varying. This remains an interesting problem for the future.

## Supporting Information

Figure S1Transition times between decay phases for HCV viral load decline after initiation of interferon-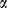 therapy. Fit of Neumann et al. model (solid line) to data (dots) from [3], with transition time calculated by maximizing the curvature 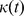 (14) (cross) of the CE model (2), for patients (a) 1B, (b) 1E, (c) 1F, (d) 2D, (e) 2E, (f) 3A, (g) 3D, and (h) 3F.(EPS)

Figure S2Approximate and analytic solution of VE model assuming sofosbuvir treatment (see Table 1 for parameters) and initial viral load of 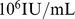. (a) Comparison of analytic solution (equation (11)) and the approximation (equation (16)). (b) Relative error in 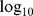 of approximation. Note the error near 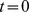 is 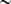
10%. (c) Comparison of analytic solution (equation (11)) and the approximation (equation (16)) with 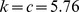 days^−1^. (d) Relative error in 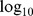 of approximation in the case 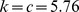 days^−1^. Note the error near 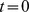 is 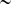
20%.(EPS)

Table S1Modified Bessel function order 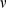 for the different treatment regimens in Table 1. 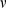 is the order of the modified Bessel functions 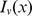 and 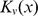 in the solution (11). The approximation to the analytic solution that we use depends on whether 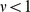 or 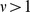 (cf. (16)).(PDF)
