# Supplementary material for: A Hepatitis C Virus Infection Model with Time-Varying Drug Effectiveness: Solution and Analysis
Source: PLoS Comput Biol. 2014 Aug 7;10(8):e1003769. doi: 10.1371/journal.pcbi.1003769 (PMC4125050; doi:10.1371/journal.pcbi.1003769)
Supplement: Table S1 — Modified Bessel function order for the different treatment regimens in Table 1. is the order of the modified Bessel functions and in the solution (11). The approximation to the analytic solution that we use depends on whether or (cf. (16)). (PDF) [file pcbi.1003769.s003.pdf]

Table S1. Modified Bessel function order ν for the different treatment regimens in Table 1. ν is the order of the modified Bessel functions $I_\nu(x)$ and $K_\nu(x)$ in the solution (11). The approximation to the analytic solution that we use depends on whether $\nu < 1$ or $\nu > 1$ (cf. (16)).

**Treatment**	$\boldsymbol{\nu}$
Telaprevir	4.48
Mericitabine, qd 750 mg, flat	5.64
Mericitabine, qd 750 mg, non-flat	26.02
Mericitabine, qd 1500 mg, flat	5.64
Mericitabine, qd 1500 mg, non-flat	26.00
Mericitabine, bid 750 mg, flat	2.94
Mericitabine, bid 750 mg, non-flat	13.90
Mericitabine, bid 1500 mg, flat	2.94
Mericitabine, bid 1500 mg, non-flat	13.90
Silibinin	2.57
Danoprevir, 100 mg	0.24
Danoprevir, 200 mg	0.24
Danoprevir, 300 mg	0.24
Sofosbuvir	0.64
